# Tribological Aspects of Slide Friction Diamond Burnishing Process

**DOI:** 10.3390/ma18194500

**Published:** 2025-09-27

**Authors:** Gyula Varga, Angelos P. Markopoulos

**Affiliations:** 1 Faculty of Mechanical Engineering and Informatics, University of Miskolc, H-3515 Miskolc, Hungary; 2 Laboratory of Manufacturing Technology, School of Mechanical Engineering, National Technical University of Athens, 15780 Athens, Greece; amark@mail.ntua.gr

**Keywords:** slide friction diamond burnishing, Abbott–Firestone curve, factorial experiment design

## Abstract

Even though the foundations of diamond burnishing as a research topic were laid long ago, numerous scientific papers still deal with examining various aspects of the burnishing process today. One such aspect is the investigation of the 3D roughness parameters related to the tribological characteristics of the machined surface, which is detailed in the present study. In this study, the positive properties of slide friction diamond burnishing are presented through the examination of surface quality, which plays a key role in tribological assessment. This study analyzed the surface layer condition of X5CrNi18-10 stainless austenitic chromium–nickel steel test pieces after burnishing. Among the finishing operations, burnishing is an economical and low-environmental impact process. The study includes a description of the technological characteristics of turning and diamond burnishing processes. The main characteristics of the Abbott–Firestone curve are described, and parameter improvement factors are introduced. The experimental results and their evaluations are presented by analyzing the values of the Abbott–Firestone surface curves. The study concludes that the best improvement ratios of *S_a_* (arithmetical mean height), *S_q_* (root mean square height), *S_z_* (maximum height) *IS_a_*, *IS_q_*, and *IS_z_* roughness improvements were achieved when using the parameter combination *v*_2_ = 55.578 m/min, *f*_2_ = 0.050 mm/rev and *F*_4_ = 50 N.

## 1. Introduction

With the advancement of the Fourth Industrial Revolution, ensuring the surface quality of precision-machined components is gaining increasing importance [1]. According to the literature, from a tribological perspective, the reliability and service life of machine elements largely depends on the outcome of the manufacturing process and the microstructure of the component’s surface [2]. The resulting surface texture has a significant impact on the wear resistance and fatigue strength of the components [3]. Conventional machining methods—such as turning and milling—are often insufficient to achieve the required surface quality; therefore, additional finishing operations, such as grinding or burnishing, are often necessary.

Meng et al. [4] reviewed the early 2020s developments in the field of tribology. Out of the 3450 articles published during this period, approximately 1000 peer-reviewed papers were selected and evaluated as key contributions to tribological research worldwide. The survey highlights advances in lubrication, wear and surface engineering, biotribology, high-temperature tribology, and computational tribology, presenting the latest findings in both fundamental and applied research. In this paper, we present the results of surface topography investigations related to tribology obtained through sliding burnishing. However, prior to that, we provide an overview of some characteristic features of selected tribological studies.

## 2. Literature Review

Burnishing is a surface improvement process based on the principle of plastic de-formation, in which the tool loads the surface irregularities to the plasticity limit and then greatly reduces the surface irregularities. Burnishing can therefore reduce surface roughness and increase hardness and residual stress, which has a positive effect on the fatigue limit [5,6]. Burnishing can be performed with ball, roller, or diamond-tipped tools, on both CNC and conventional machines [7]. Diamond burnishing in particular has proven to be an effective chip-free process that is suitable for treating flat, cylindrical, and complex surfaces [8]. However, the effect of the process parameters (force, feed rate, speed) has not yet been accurately modelled in all cases.

Several comprehensive studies have been published in recent years. A previous re-view paper by Maximov and Duncheva [9] summarized the research on diamond burnishing from 2019 to 2023. The study presents in detail the different processes that can be applied to both external and internal cylindrical surfaces, as well as to flat and complex surfaces. The main goal of the method is to increase wear resistance and fatigue strength, which is confirmed by both theoretical models and practical tests. The directions of the research are clear, but the exact definition of the parameters for industrial application remains an open question. In a 2024 study [10], Maximov and Duncheva investigated the effects of cryogenic and cold burnishing on the surface integrity and operational performance of metals. Temperatures below −180 °C induce structural transformations that have a beneficial effect on lifetime. Cryogenic treatment can be used as a stand-alone or additional operation and is an environmentally friendly alternative. The review pays special attention to hybrid processes, where burnishing is performed under cryogenic conditions. Although the results are encouraging, the cost and technological barriers of the method currently limit its widespread adoption.

In a 2025 study [11], Maximov and Duncheva reviewed the development of diamond burnishing between 1962 and 2025. They discussed traditional, ultrasonic, minimum quantity lubrication (MQL), and combined processes, as well as the application of finite element simulations. The review highlighted that although FEM models help us to understand the processes, they often do not accurately reflect real tribological behavior. Although the development directions are clear, the discrepancies between simulations and experimental results require further research.

Various investigations have focused on treatments improving wear. In the analysis of tribological effects, Korzynski et al. [12] investigated the effect of diamond coating on valve stems, where both hardness and residual stress developed favorably. According to their results, surface improvement also increased the service life of engine components. Sedlaček et al. [13] demonstrated the predictability of friction behavior based on the *S_sk_* (skewness) and *S_ku_* (kurtosis) parameters of surface roughness. Shuster et al. [14] used 17 surface texture parameters to evaluate density and wear during alternating motion. Prajapati and Tiwari [15] emphasized the role of the *S_q_* (root mean square height), *S_dq_* (root mean square slope), and *V_vv_* (dale void volume) parameters. Sedlaček et al. [16] reported that the *S_ku_* (kurtosis) and *S_sk_* (skewness) parameters showed a strong correlation with the tribological properties of mating surfaces. According to Reddy et al. [17], there is a close correlation between the *V_vv_* (dale void volume) and *S_q_* (root mean square height) parameters and the degree of wear. Similarly, a study by Shi et al. [18] found a close correlation between the *V_vv_* (dale void volume) and *V_vc_* (core void volume) parameters and the degree of wear. Although the multitude of parameters allows for more accurate characterization, their validation and standardization in industrial practice is still lacking.

Lavrys et al. [19] demonstrated on porous titanium materials that ball peening in-creases wear resistance, while Dzierwa et al. [20] examined roll peening on steel test specimens. According to the results of Dzierwa et al, load is the most important factor directly influencing surface roughness and hardness. However, these studies are based on short-term wear tests, so long-term durability is less well documented.

Swirad [21] demonstrated a significant reduction in roughness and wear during ball rolling of Ti6Al4V titanium alloy, while other studies reported a friction reduction of more than 40% in steel samples. However, the improvement was not always clear in high-temperature tests. This indicates that the effect of heat is a key factor that requires further detailed investigation.

Grudzien’s study [22] pointed out that not only residual stress but also the geometry of the surface topography determines tribological performance. Dzierwa and Markopoulos [23] investigated ball peening of hardened steel in a CNC environment and emphasized that appropriate feed rate and force significantly improve surface roughness. However, the optimal parameters are still not uniformly defined at the industrial level.

Varga et al. [24] investigated the treatment of titanium alloys produced by selective laser melting (SLM) using sliding friction burnishing. According to their results, burnishing significantly improved surface integrity, but the effect was highly dependent on the parameters of 3D printing using selective laser melting. This highlights the strong interaction between selective laser melting and surface treatment, which has not yet been fully explored at the industrial level.

Swirad and Pawlus [25,26,27,28] conducted several studies comparing dry and lubricated ball bearings. They found that when lubrication was applied, friction and wear were reduced by more than 40%. However, in high-temperature tests, a positive effect was not always observed. This suggests that the interaction between lubrication and heat effects is a key issue for future research.

Torres et al. [29] studied the effect of microstructure on stainless steels and concluded that changes in the friction coefficient are more significant than changes in material structure. Velázquez-Corral et al. [30] used ultrasonic assistance to strengthen residual stresses, while Kuznetsov et al. [31] increased the hardness of AISI 304 steel using nanostructured burnishing. These processes are promising, but their complexity limits their application in industrial settings.

Bednarski et al. [32] conducted friction burnishing experiments on composite materials, confirming a significant increase in microhardness. The results showed that the degree of improvement depends largely on the composition of the material. However, the long-term behavior of heterogeneous composites has yet to be fully explored.

Abbott–Firestone curves are widely used to describe surface roughness, especially after cutting [33,34,35] and burnishing operations [36,37]. Tomov et al. [33] and Molnár and Sztankovics [35] investigated the tribological significance of the parameters, while Skoczylas and Kłonica [36] highlighted the effect of lubricants. Kubatova and Melichar [37] confirmed that the parameters of the curve are independent of software filters. Although the method is reliable, it is not yet uniformly applied in industrial quality control between manufacturing processes.

Overall, the literature clearly shows that burnishing processes have a positive effect on surface integrity, reduce wear, and increase fatigue life. However, several factors—such as heat effects, lubrication, parameter optimization, and the long-term tribological behavior of different materials—require further research. The challenge for the future is to develop cost-effective burnishing processes that can be applied stably on an industrial scale.

**Figure 1:**
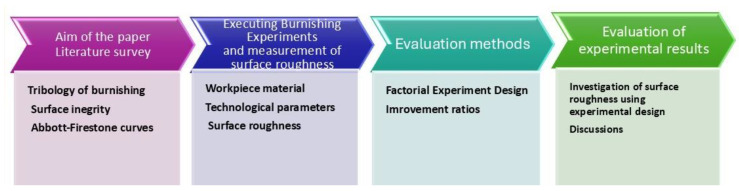
Structure of this paper.

Figure 1 shows the structure of this paper.

## 3. Materials and Methods

Burnishing is a cold plastic deformation technique commonly applied for the finishing of external cylindrical surfaces. The process offers several benefits, such as reduced surface roughness, enhanced microhardness, the introduction of compressive residual stress, improved dimensional precision, and environmental advantages, as it requires minimal use of lubricants [38].

**Figure 2:**
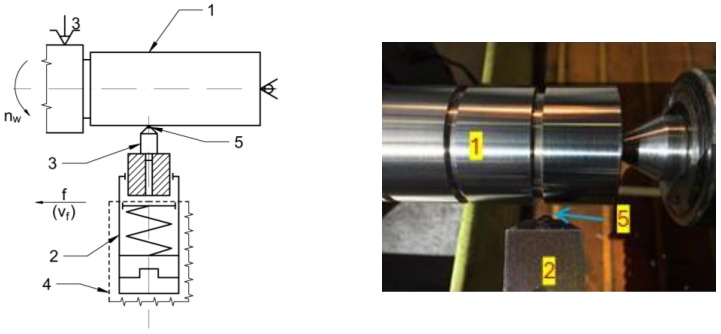
Schematic representation of diamond burnishing process [39]. 1—workpiece, 2—tool body, 3—burnishing insert, 4—toolholder, 5—diamond tip.

Figure 2 [39] illustrates the principle of diamond burnishing, where a spherical-tipped diamond tool is pressed against the surface of a rotating workpiece under controlled parameters and force.

The aim of our research is to investigate the surface roughness produced by the diamond burnishing process. To ensure comparable results, uniform test specimens were prepared on machine tools located in the workshop of the Institute of Manufacturing Science of the University of Miskolc. The geometric characteristics of the test specimens used for the burnishing experiments were as follows: five consecutive cylindrical surfaces with a diameter of Ø47.20 mm and a length of 26 mm. There was 5 mm wide grooving between the cylindrical parts. The turning technological parameters were kept uniform across all specimens:

$$v_{c\;turning}=82.8\;m/min;\;f_{turning}=0.05\;mm/rev$$

This uniformity allowed us not only to examine the roughness of the surface after diamond burnishing but also to quantify the extent of surface improvements specifically, the enhancement in surface quality compared to the original turned surface. This comparison is particularly important, as identical turning parameters do not always yield identical surface roughness outcomes.

The material of the specimen is X5CrNi18-10 (PROFILINOX S.p.A., Parma, Italy) austenitic stainless chromium–nickel steel alloy. This material grade is one of the most widely utilized stainless steels due to its exceptional corrosion resistance, excellent cold formability, and good weldability. It exhibits notable resistance to dilute acids, freshwater, and atmospheric moisture, making it suitable for a broad range of industrial applications. This material is commonly employed in the automotive industry, particularly in vehicle manufacturing, as well as in mechanical and equipment engineering, where it is used for components such as machine parts and pump elements. A key advantage of X5CrNi18-10 is its high resistance to intergranular corrosion, a property that can be further enhanced through surface modification techniques such as diamond burnishing. The chemical composition of X5CrNi18-10 is provided in Table 1 [40], while its mechanical properties are listed in Table 2 [40].

The physical properties of X5CrNi18-10 stainless steel at 20 °C are as follows [40]: density—7.9 g/cm^3^, specific heat capacity—500 J/kg·K, thermal conductivity—15 W/m·K, and electrical resistivity—0.73 Ω·mm^2^/m.

The burnishing experiments were carried out on a refurbished and structurally rigid EU-400/01 SZIM lathe (Machine Tool Works, Budapest, Hungary). A polycrystalline diamond (PCD) burnishing tool with a tip radius of *R* = 3.5 mm was employed. Lubrication was applied manually using SAE 15W-40 oil (Motorex, Bützberg, Switzerland) in a quantity of Qₒ *=* (5 ± 1) × 10^−6^ m^3^.

The technological parameters of diamond burnishing were combined using two feed rates, two spindle speeds, and four burnishing forces. The independent parameter combinations are given in Table 3. 

Feed rate:

$$f_{1}=0.0125\;mm/rev;\;f_{2}=0.05\;mm/rev;\;f_{3}=0.1\;mm/rev$$

Workpiece RPM:

$$n_{1}=265\times 1/min;\;n_{2}=375\times 1/min$$

Burnishing force (the force set directly on the machine tool via spring preload):

$$F_{1}=20\;N;\;F_{2}=30\;N;\;F_{3}=40\;N;\;F_{4}=50\;N$$

The burnishing speed can be calculated by Equation (1):

(1) $$v=D\cdot \pi \cdot n\;\left[m/min\right]$$

where *D* is the diameter of the specimen.

In this work, surface roughness was evaluated through multiple metrics, including three-dimensional roughness parameters. The measurements were performed using an AltiSurf 520 surface metrology system, with data processing carried out in AltiMap software (version 6.2) supplied with the instrument. Detailed technical specifications for the AltiSurf 520 are available in [41]. The system operates on a non-contact, optical measurement principle, incorporating confocal chromatic, interferometric, and other techniques. Its measurement range extends from 100 µm to 25 mm, with a vertical (Z) resolution of up to 2 nm and a lateral resolution of 0.7 µm. Step-height accuracy for a 1 µm standard is 0.005%. The lowest measurable roughness (Ra/Sa) is 20 nm/20 nm, in compliance with ISO 21920-2 [42] and ISO 25178 **[43]**. All measurements were conducted in the metrology laboratory of the Institute of Manufacturing Science at the University of Miskolc, under consistent laboratory conditions and identical instrument settings. Environmental factors such as temperature and humidity were controlled to reduce measurement uncertainty and ensure reproducibility of results.

## 4. Measurement Results

This chapter contains the results of the examination of 3D Surface Roughness Parameters, the use of full Factorial Experiment Design analysis for the examination of functional volume area roughness parameters, and the examination of functional volume area roughness parameters for the case when workpiece feed is constant.

### 4.1. Examination of 3D Surface Roughness Parameters

After executing the experiments and measuring the 3D surface roughness parameters we summarized the measured data to Table 4. Table 4 contains the measured 3D surface roughness parameters (arithmetical mean height (*S_a_*), root mean square height (*S_q_*), maximum height (*S_z_*), maximum peak height (*S_p_*), maximum pit depth (*S_v_*), skewness (*S_sk_*), kurtosis (*S_ku_*)) for parameter variations V1–V16, before and after burnishing.

The enhancement of surface roughness achieved through diamond burnishing was evaluated using so-called improvement factors. Since the surface condition and corresponding roughness parameters after turning (i.e., prior to diamond burnishing) can vary, establishing the initial state is essential for accurately assessing the effect of the burnishing process. The improvement factor was determined according to Equation (2). This equation represents a general formulation that can be applied to various surface roughness parameters, where “I” denotes the improvement, “X” corresponds to “S” for 3D parameters, and “y” serves as a placeholder for different types of roughness metrics (e.g.: y can be “a”, “q” and “z”).

(2) $${IX}_{y}=\frac{X_{y;after\;burnishing}-X_{y;before\;burnishing}}{X_{y;before\;burnishing}}\cdot 100\;\left[\%\right]$$

The measured 3D surface roughness parameters and the calculated improvement ratios for the *F*_1_–*F*_4_; *F*_2_–*F*_4_, and *F*_3_–*F*_4_ burnishing force variants are contained in Table A1.

**Figure 3:**
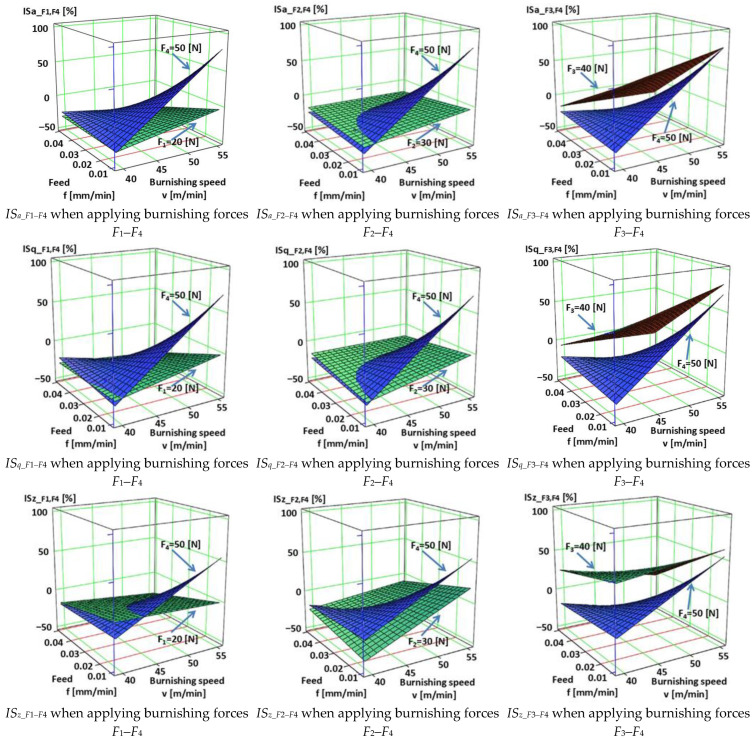
Change in surface roughness improvement ratio for different parameter combinations.

The empirical Equations (3)–(11) relate to *IS_a_*, *IS_q_*, and *IS_z_* when comparing the results belonging to *F*_1_–*F*_4_, *F*_2_–*F*_4_, and *F*_3_–*F*_4_ burnishing forces. They were determined on the base of Factorial Experiment Design [44] with MathCAD 15 and are illustrated in Figure 3.

(3) $${IS}_{a\_F1-F4}=53.7326-2.151v-1.554\times 10^{3}f-9.325F+37.016v\cdot f+0.235v\cdot F+204.671f\cdot F-5.051v\cdot f\cdot F$$

$${IS}_{a\_F1-F4}\;\to R^{2}=0.999954264$$

(4) $${IS}_{a\_F2-F4}=385.721-10.175v-9.33\times 10^{3}f-15.965F+240.683v\cdot f+0.395v\cdot F+360.19f\cdot F-9.124v\cdot f\cdot F$$

$${IS}_{a\_F2-F4}\;\to R^{2}=0.999659502$$

(5) $${IS}_{a\_F3-F4}=1.464\times 10^{3}-24.84v-3.212\times 10^{4}f-37.52F+577.362v\cdot f+0.689v\cdot F+816.0f\cdot F-15.858v\cdot f\cdot F$$

$${IS}_{a\_F3-F4}\;\to R^{2}=0.999257148$$

(6) $${IS}_{q\_F1-F4}=64.7205-2.154v-1.958\times 10^{3}f-9.098F+42.969v\cdot f+0.224v\cdot F+214.553f\cdot F-5.191v\cdot f\cdot F$$

$${IS}_{q\_F1-F4}\;\to R^{2}=0.999752488$$

(7) $${IS}_{q\_F2-F4}=284.083-7.986v-8.013\times 10^{3}f-13.485F+215.248v\cdot f+0.34v\cdot F+335.654f\cdot F-8.636v\cdot f\cdot F$$

$${IS}_{q\_F2-F4}\;\to R^{2}=0.999681126$$

(8) $${IS}_{q\_F3-F4}=1.317\times 10^{3}-21.39v-2.928\times 10^{4}f-34.144F+530.51v\cdot f+0.608v\cdot F+760.9f\cdot F-14.942v\cdot f\cdot F$$

$${IS}_{q\_F3-F4}\;\to R^{2}=0.999493478$$

(9) $${IS}_{z\_F1-F4}=202.772-4.458v-4.501\times 10^{3}f-9.317F+92.363v\cdot f+0.216v\cdot F+220.154f\cdot F-5.218v\cdot f\cdot F$$

$${IS}_{z\_F1-F4}\;\to R^{2}=0.99945485$$

(10) $${IS}_{z\_F2-F4}=-149.581+1.524v-1.887\times 10^{3}f-2.27F+77.096v\cdot f+0.097v\cdot F+167.975f\cdot F-4.913v\cdot f\cdot F$$

$${IS}_{z\_F2-F4}\;\to R^{2}=0.999594253$$

(11) $${IS}_{z\_F3-F4}=930.79-15.58v-1.416\times 10^{4}f-23.877F+300.075v\cdot f+0.439v\cdot F+413.318f\cdot F-9.372v\cdot f\cdot F$$

$${IS}_{z\_F3-F4}\;\to R^{2}=0.999882163$$

### 4.2. Full Factorial Experiment Design Analysis of Functional Volume Area Roughness Parameters

**Figure 4:**
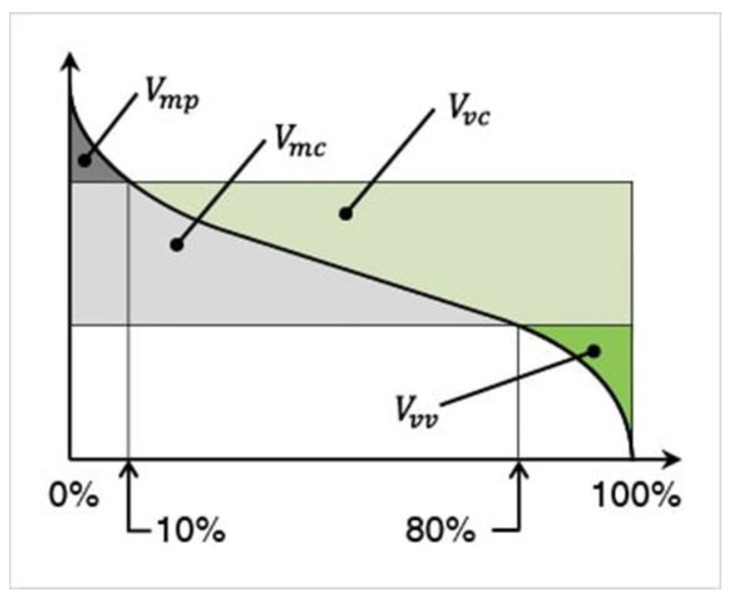
Main volume parameters on Abbott–Firestone curve: *V_mp_* (peak material volume), *V_mc_* (core material volume), *V_vc_* (core void volume), and *V_vv_* (dale void volume) [43].

The Abbott–Firestone curve (Figure 4) can also be used for characterization of “surface structure”. In this case, four main volumetric characteristics can be distinguished: *V_mp_* (peak material volume), *V_mc_* (core material volume), *V_vc_* (core void volume), and *V_vv_* (dale void volume). To use volume parameters, it is necessary to specify the material ratio values that separate the reduced peaks and reduced dales from the core surface. By default, p = 10% and q = 80% values are used. *V_mc_* (core material volume) is the volume of material contained within the “core” layer, i.e., between the 10% and 80% levels of the cumulative height distribution. In other words, if all measured points are sorted by height, *V_mc_* is the volume of the “core” mass of the surface. *V_mp_* (peak material volume) refers to the material volume at a p% material ratio. *V_vc_* (core void volume) is the difference between the void volume corresponding to the p% surface material ratio and the void volume corresponding to the q% surface material ratio. *V_vv_* (dale void volume) refers to the dale void volume at a p% surface material ratio [43].

**Figure 5:**
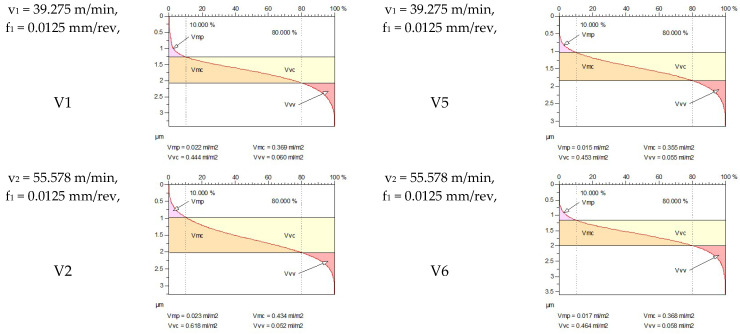

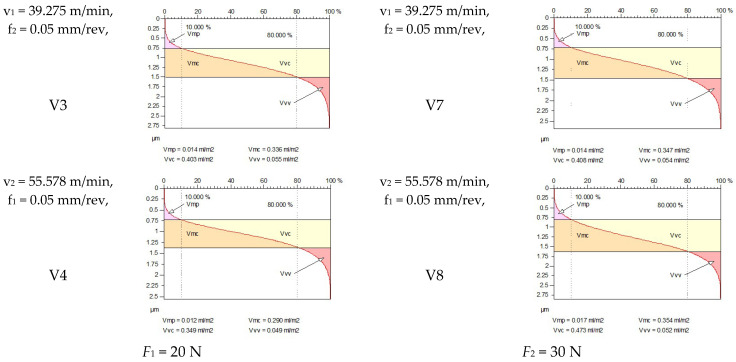
Volume parameters calculated on areal material curve using default material threshold (10% and 80%) when *F*_1_ = 20 N and *F*_2_ = 30 N burnishing forces were applied.

Figure 5 shows the volume parameters calculated on the areal material curve and using default material threshold (10% and 80%) when *F*_1_ = 20 N and *F*_2_ = 30 N burnishing forces were applied. Figure 5 shows the same when *F*_3_ = 40 N and *F*_4_ = 50 N burnishing forces were applied. The V1–V16 signs in Figure 4 and Figure 5 relate to the applied technological parameters in Table 3.

**Figure 6:**
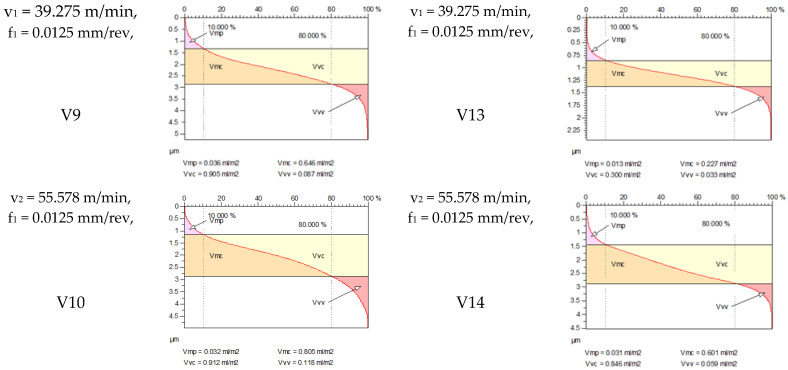

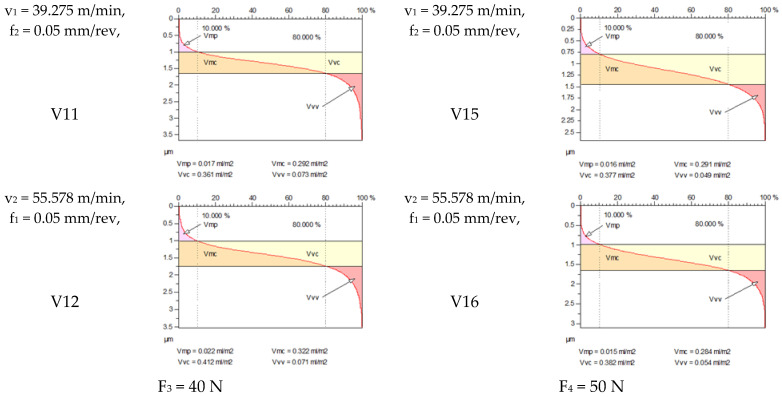
Volume parameters calculated on areal material curve using default material threshold (10% and 80%) when *F*_3_ = 40 N and *F*_4_ = 50 N burnishing forces were applied.

Figure 5 and Figure 6 contain the values of the volume parameters, which values are summarized in Table A2. Tribological evaluation of the values in Table A2 are as follows. We create a ratio, Equation (12) which has tribological meaning.

(12) $$\frac{{DV}_{mc}}{{DV}_{vc}}=\frac{V_{mc}-{V_{mc}}^{\prime }}{V_{vc}-{V_{vc}}^{\prime }}$$

where *DV_mc_* is the difference between the value of core material volume after and before burnishing and *DV_vc_* is the difference between the value of core void volume after and before burnishing

The *DV_mc_*/*DV_vc_* ratio gives the ratio of the surface area to its “core void”/“void capacity”, i.e., how much (core) material (load-bearing zone) is available compared to the volume of the core cavity (lubricant storage). Tribologically, this is an important indicator of the compromise between load capacity, lubricant storage, and mixed/hydrodynamic operation. High *DV_mc_*/*DV_vc_* a lot of material in the core zone, little void capacity which indicates good dry/asperity load capacity, less lubricant storage. This is advantageous if the primary goal is load-bearing and minimal slip, but is disadvantageous due to weaker oil film formation and faster wear if lubrication is insufficient. Low *DV_mc_*/*DV_vc_* means relatively high void capacity: good lubricant storage and debris collection, favorable for the development of hydrodynamic/in-field lubrication, but with the disadvantages of lower initial load capacity and higher asperity local stress. This illustrates the rationale for examining the *DV_mc_*/*DV_vc_* ratio.

**Figure 7:**
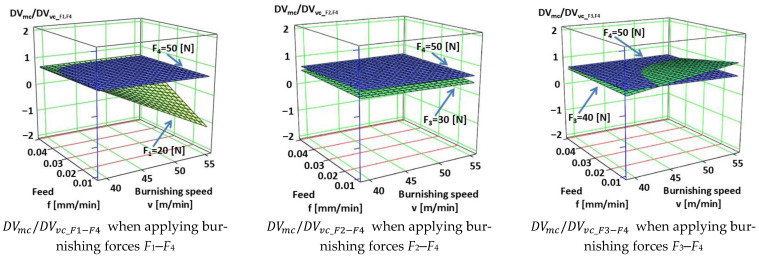
Changes in *DV_mc_/DV_vc_* ratio for different parameter combinations.

The empirical Equations (13)–(15) relating to *DV_mc_*/*DV_vc_* for *F*_1_–*F*_4_, *F*_2_–*F*_4_, and *F*_3_–*F*_4_ burnishing forces were determined on the base of Factorial Experiment Design [44] with MathCAD 15 and are illustrated in Figure 7.

(13) $${{DV}_{mc}/DV}_{vc\_F1-F4}=11.046-0.26v-208.118f-0.2F+5.224v\cdot f+5.051\times 10^{-3}v\cdot F+3.979f\cdot F-0.101v\cdot f\cdot F$$

$${{DV}_{mc}/DV}_{vc\_F1-F4}\;\to R^{2}=0.999410262$$

(14) $${{DV}_{mc}/DV}_{vc\_F2-F4}=0.4661-0.01v-15.483f-0.012F+0.51v\cdot f+0.0001v\cdot F+0.1258f\cdot F-6.87\times 10^{-3}v\cdot f\cdot F$$

$${{DV}_{mc}/DV}_{vc\_F2-F4}\;\to R^{2}=1.0$$

(15) $${{DV}_{mc}/DV}_{vc\_F3-F4}=-10.48+0.259v+223.7f+0.231F-5.401v\cdot f-5.411\times 10^{-3}v\cdot F-4.66f\cdot F+0.1116v\cdot f\cdot F$$

$${{DV}_{mc}/DV}_{vc\_F3-F4}\;\to R^{2}=1.0$$

### 4.3. Examination of Functional Volume Area Roughness Parameters When Workpiece Feed Is Constant

Table A3 contains the measured values of functional volume area roughness parameters *V_mp_* (peak material volume), *V_mc_* (core material volume), *V_vc_* (core void volume), and (*V_vv_* (dale void volume) for parameter variations F1–F8, before and after burnishing. In these examinations the burnishing feed was constant (*f*_3_ = 0.1 mm/rev). Other technological parameters can be seen in Table A3.

Similarly to the improvement factors for the 3D roughness parameters presented in Equation (2), the formula can also be written for the improvement of functional volume area roughness parameters (*V_mp_*, *V_mc_*, *V_vc_*, and *V_vv_*). The improvement factor was determined (similarly to Equation (1)) according to Equation (16). This equation represents a general formulation that can be applied to various surface roughness parameters, where “I” denotes the improvement, “Y” corresponds to “ V (different functional volume area roughness parameters)”, and “y” serves as a placeholder for different types of roughness metrics (e.g.,: y can be “mp”, “mc”, “vc” or “vv”).

(16) $${IY}_{y}=\frac{Y_{before\;burnishing}-Y_{after\;burnishing}}{Y_{before\;burnishing}}\cdot 100\;\left[\%\right]$$

**Figure 8:**
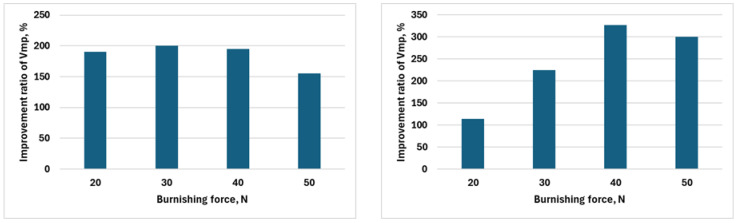

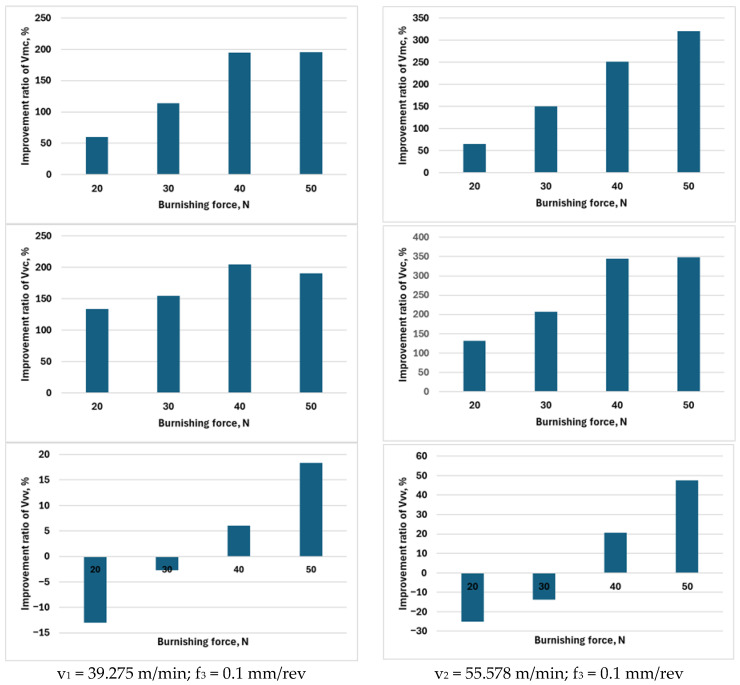
Improvement ratios of *V_mp_*, *V_mc_*, *V_vc_*, and *V_vv_* for the two burnishing speeds applied.

Figure 8 displays the improved ratios of the measured values of functional volume area roughness parameters *V_mp_* (peak material volume), *V_mc_* (core material volume), *V_vc_* (core void volume), and (*V_vv_* (dale void volume) for parameter variations F1–F8, before and after burnishing for *F*_1_–*F*_4_ burnishing forces and *v*_1_–*v*_2_ burnishing speeds.

## 5. Discussion

After examining the results of the research, it can be stated that Figure 3 shows the improvement factors of the 3D roughness parameters *S_a_*, *S_q_*, and *S_z_* due to burnishing, considering the burnishing force pairs *F*_1_–*F*_4_, *F*_2_–*F*_4_, and *F*_3_–*F*_4_. After studying Table A1 and Figure 3, the following can be observed. When applying a burnishing force of *F*_1_ = 20 N, the greatest improvement in terms of the improvement parameters *IS_a_*, *IS_q_*, and *IS_z_* is achieved at higher speed (*v*_2_ = 55.578 m/min) and higher feed (*f*_2_ = 0.050 mm/rev). Interestingly, when applying a burnishing force of *F*_2_ = 30 N, the lower speed (*v*_1_ = 39.275 m/min) and lower feed (*f*_1_ = 0.0125 mm/rev) parameter pair provides greater roughness improvement for all the three examined 3D roughness parameters. It should be noted that when applying a burnishing force of *F*_2_ = 30 N, the *IS_z_* improvement factor changes sign, i.e., the surface does not improve due to burnishing, but deteriorates compared to before burnishing when applying higher speed (*v*_2_ = 55.578 m/min) and lower feed (*f*_1_ = 0.0125 mm/rev) values. In the case when the burnishing force is *F*_3_ = 40 N, the higher burnishing speed (*v*_2_ = 55.578 m/min) and the higher feed (*f*_2_ = 0.050 mm/rev) also provided the highest improvement ratio values. It can also be noticed that at the lower feed (*f*_1_ = 0.0125 mm/rev) both speeds already showed surface roughness deterioration. When applying the lower speed (*v*_1_ = 39.275 m/min) and the higher feed (*f*_2_ = 0.050 mm/rev), there was still an improvement in the *IS_a_* and *IS_q_* values, but a deterioration in the *IS_z_* value. When applying the burnishing force of *F*_4_ = 50 N, the higher feed (f_2_ = 0.050 mm/rev) and both speeds caused roughness improvement in terms of *IS_a_*, *IS_q_*, and *IS_z_*. This was observed even when using lower speed (*v*_1_ = 39.275 m/min) and lower feed (*f*_1_ = 0.0125 mm/rev).

Table A2 and Figure 7 deal with the analysis of the *DV_mc_*/*DV_vc_* ratio. Figure 7 shows the change in the *DV_mc_*/*DV_vc_* ratios due to burnishing, considering the burnishing force pairs *F*_1_–*F*_4_, *F*_2_–*F*_4_, and *F*_3_–*F*_4_. The *DV_mc_*/*DV_vc_* ratio shows how much the volume of material in the core zone is compared to the volume of the voids. If *DV_mc_* is large, there is a lot of material in the core zone (more compact surface, less lubricant storage capacity). If *DV_vc_* is large, there are many voids in the core zone (more lubricant storage capacity, but less carrier surface). According to Figure 7, when the chosen technological parameters are applied, the *DV_mc_*/*DV_vc_* ratio is predominantly (with one exception) less than 1. This indicates that the core zone is cavity-dominant, i.e., the surface is characterized from a tribological point of view by the fact that the surface can store a relatively large amount of lubricant. Its advantage is good lubrication retention and friction and wear reduction. The disadvantage is that as the actual bearing surface is smaller, the pressure can be concentrated on the remaining material surfaces, which can lead to microplastic deformation.

Table A3 and the corresponding Figure 8 show changes in the improvement factors of the various parameters of the Abbott–Firestone curve due to burnishing change depending on the burnishing. Figure 8 contains the values for the lower speed (*v*_1_ = 39.275 m/min) and the higher speed (*v*_2_ = 55.578 m/min) in a separate column. The *IV_mc_* value related to the core zone at the lower speed (*v*_1_ = 39.275 m/min) when *F*_1_ = 20 N, *F*_2_ = 30 N, and *F*_3_ = 40 N are applied increases almost linearly from 60.21% to 194.72%; however, when *F*_4_ = 50 N is applied, the further increase is minimal, i.e., the improvement value is 195.5%. Therefore, in the investigated burnishing force range, it is not worth increasing it towards *F*_3_ = 40 N. When using a higher speed (*v*_2_ = 55.578 m/min), the *IV_mc_* improvement factor increases in line with the previous trend even when setting the burnishing force *F*_4_ = 50 N. When examining the *IV_vc_* improvement factor related to the lubricant retention ability, very similar conclusions can be drawn to the previous ones. When using a lower burnishing speed (*v*_1_ = 39.275 m/min), it is also advisable to increase the burnishing force up to a value of *F*_3_ = 40 N, while at a higher burnishing speed (*v*_2_ = 55.578 m/min), the burnishing force can be increased up to a value of *F*_4_ = 50 N. It is true that the difference in the improvement factor is minimal when using the burnishing forces *F*_3_ = 40 N and *F*_4_ = 50 N.

Since the numerical values of four additional 3D surface roughness parameters (maximum peak height (*S_p_*), maximum pit depth (*S_v_*), skewness (*S_sk_*), kurtosis (*S_ku_*)) have been determined (Table 4), our future goal is to examine how these parameters depend on the burnishing technological parameters. Furthermore, we intend to measure and document the surface roughness of the diamond burnishing tool before, during and after the execution of the diamond burnishing experiments. Our further goal is to expand the ranges of burnishing technological parameters, such as feed and burnishing speed.

## 6. Conclusions

Based on the conducted investigations, it can be stated that the burnishing force, as well as the other two parameters (speed and feed), have a significant effect on the improvement of the 3D roughness characteristics (*IS_a_*, *IS_q_*, *IS_z_*). The most favorable surface quality improvement was generally observed at higher feed and speed values; however, in certain parameter combinations—particularly at higher speed and lower feed—surface deterioration also occurred. The results highlight that the optimal parameters of burnishing depend not only on the magnitude of the force but also on the interaction between speed and feed. Overall, selecting the appropriate parameter pairs is essential for effectively reducing surface roughness. The best *IS_a_*, *IS_q_*, and *IS_z_* surface roughness improvements were obtained with the following parameter combination:

*v*_2_ = 55.578 m/min, *f*_2_ = 0.050 mm/rev, and *F*_4_ = 50 N.

According to the results, the value of the *DV_mc_*/*DV_vc_* ratio was predominantly below 1, which indicates a cavity-dominant core zone. From a tribological point of view, this is advantageous due to good lubricant retention capacity and reduced friction, but the smaller actual bearing surface may lead to increased pressure concentration and microplastic deformation. An exception was observed with the combination of *F*_3_ = 40 N force, higher speed (*v*_2_ = 55.578 m/min), and lower feed (*f*_1_ = 0.0125 mm/rev), where the ratio exceeded 1, while under certain conditions vibrations occurred during the experiment, making the measurement results invalid. Overall, the technological parameters of burnishing fundamentally determine the tribological properties of the surface.

The improvement factors of the Abbott–Firestone curve parameters showed that at lower speed (*v*_1_ = 39.275 m/min), it is advisable to increase the burnishing force only up to *F*_3_ = 40 N, since further increase results in negligible improvement. At higher speed (*v*_2_ = 55.578 m/min), improvement continues even at *F*_4_ = 50 N, although the difference between *F*_3_ = 40 N and *F*_4_ = 50 N is minimal. This indicates that the optimal force limit depends on the applied speed, but excessive force increase does not lead to proportional surface quality improvement. In summary, the efficiency of burnishing can be economically maximized by selecting the proper combination of speed and force.

## Figures and Tables

**Table 1:** Chemical composition (wt.%) of austenitic chromium–nickel stainless steel X5CrNi18-10 [40].

C %	Si %	Mn %	P %	S %	Cr %	Ni %	N %
≤0.07	≤1.00	≤2.00	≤0.045	≤0.015	17.5–19.5	8.00–10.5	≤0.11

**Table 2:** Mechanical properties of austenitic chromium–nickel stainless steel X5CrNi18-10 at 20 °C [40].

Hardness HB 30	0.2% Yield Strength, Rp	Tensile Strength, Rm	Elongation A5	Modulus of Elasticity
HB	N/mm^2^	N/mm^2^	%	kN/mm^2^
≤215	≥190	500–700	≥45/35	200

**Table 3:** Diamond burnishing parameter variations.

	No.	Burnishing Parameters			
		n	v	f	F
		[1/min]	[m/min]	[mm/rev]	[N]
V1	1_1	265	39.275	0.0125	20
V2	1_2	375	55.578	0.0125	20
V3	1_3	265	39.275	0.0500	20
V4	1_4	375	55.578	0.0500	20
V5	2_1	265	39.275	0.0125	30
V6	2_2	375	55.578	0.0125	30
V7	2_3	265	39.275	0.0500	30
V8	2_4	375	55.578	0.0500	30
V9	3_1	265	39.275	0.0125	40
V10	3_2	375	55.578	0.0125	40
V11	3_3	265	39.275	0.0500	40
V12	3_4	375	55.578	0.0500	40
V13	4_1	375	39.275	0.0125	50
V14	4_2	375	55.578	0.0125	50
V15	4_3	265	39.275	0.0500	50
V16	4_4	375	55.578	0.0500	50
F1	7_1	265	39.275	0.1000	20
F2	7_2	375	55.578	0.1000	30
F3	7_3	265	39.275	0.1000	40
F4	7_4	375	55.578	0.1000	50
F5	8_1	265	39.275	0.1000	20
F6	8_2	375	55.578	0.1000	30
F7	8_3	265	39.275	0.1000	40
F8	8_4	375	55.578	0.1000	50

**Table 4:** Measured 3D surface roughness parameters for parameter variations V1–V16.

Before Burnishing	After Burnishing
V1	**v_1_ = 39.275 m/min, f_1_ = 0.0125 mm/rev, F_1_ = 20 N**
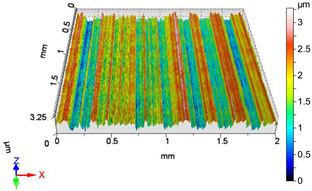	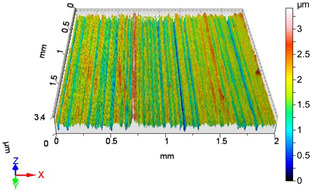
S_a_ = 0.490 µm, S_q_ = 0.585 µm, S_z_ = 3.277 µm, S_p_ = 1.588 µm, S_v_ = 1.689 µm, S_sk_ = −0.037, S_ku_ = 2.269	S_a_ = 0.331 µm, S_q_ = 0.428 µm, S_z_ = 3.414 µm, S_p_ = 1.736 µm, S_v_ = 1.678 µm, S_sk_ = −0.264, S_ku_ = 3.737
V2	**v_2_ = 55.578 m/min, f_1_ = 0.0125 mm/rev, F_1_ = 20 N**
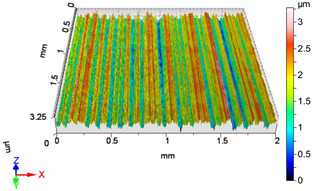	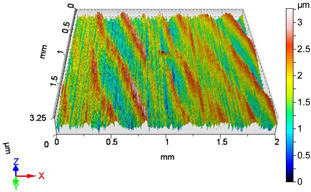
S_a_ = 0.402 µm, S_q_ = 0.493 µm, S_z_ = 3.278 µm, S_p_ = 1.567 µm, S_v_ = 1.711 µm, S_sk_ = −0.137, S_ku_ = 2.652	S_a_ = 0.386 µm, S_q_ = 0.486 µm, S_z_ = 3.265 µm, S_p_ = 1.611 µm, S_v_ = 1.654 µm, S_sk_ = 0.111, S_ku_ = 2.928
V3	**v_1_ = 39.275 m/min, f_2_ = 0.05 mm/rev, F_1_ = 20 N**
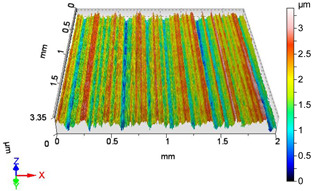	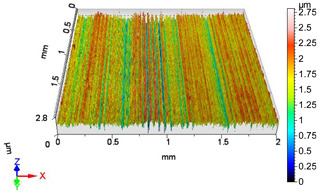
S_a_ = 0.435 µm, S_q_ = 0.535 µm, S_z_ = 3.400 µm, S_p_ = 1.560 µm, S_v_ = 1.839 µm, S_sk_ = −0.150, S_ku_ = 2.638	S_a_ = 0.298 µm, S_q_ = 0.380 µm, S_z_ = 2.817 µm, S_p_ = 1.198 µm, S_v_ = 1.619 µm, S_sk_ = −0.606, S_ku_ = 3.634
V4	**v_2_ = 55.578 m/min, f_2_ = 0.05 mm/rev, F_1_ = 20 N**
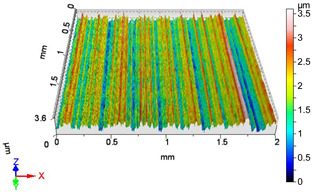	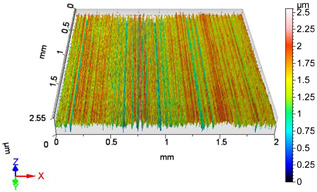
S_a_ = 0.446 µm, S_q_ = 0.557 µm, S_z_ = 3.603 µm, S_p_ = 1.835 µm, S_v_ = 1.768 µm, S_sk_ = 0.062, S_ku_ = 2.934	S_a_ = 0.258 µm, S_q_ = 0.330 µm, S_z_ = 2.556 µm, S_p_ = 1.101 µm, S_v_ = 1.455 µm, S_sk_ = −0.598,S_ku_ = 3.612
V5	**v_1_ = 39.275 m/min, f_1_ = 0.0125 mm/rev, F_2_ = 30 N**
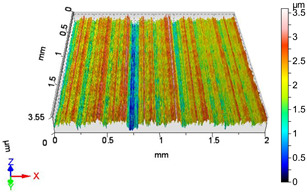	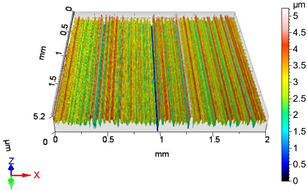
S_a_ = 0.419 µm, S_q_ = 0.563 µm, S_z_ = 5.153 µm, S_p_ = 3.222 µm, S_v_ = 1.931 µm, S_sk_ = 0.958, S_ku_ = 6.610	S_a_ = 0.315 µm, S_q_ = 0.400 µm, S_z_ = 3.144 µm, S_p_ = 1.520 µm, S_v_ = 1.625 µm, S_sk_ = −0.377, S_ku_ = 3.223
V6	**v_2_ = 55.578 m/min, f_1_ = 0.0125 mm/rev, F_2_ = 30 N**
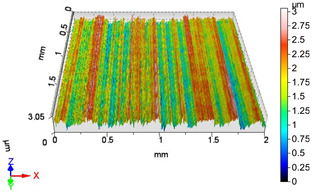	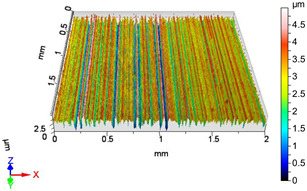
S_a_ = 0.343 µm, S_q_ = 0.428 µm, S_z_ = 3.019 µm, S_p_ = 1.308 µm, S_v_ = 1.710 µm, S_sk_ = −0.127, S_ku_ = 2.943	S_a_ = 0.329 µm, S_q_ = 0.421 µm, S_z_ = 3.588 µm, S_p_ = 1.660 µm, S_v_ = 1.928 µm, S_sk_ = −0.474, S_ku_ = 3.620
V7	**v_1_ = 39.275 m/min, f_2_ = 0.05 mm/rev, F_2_ = 30 N**
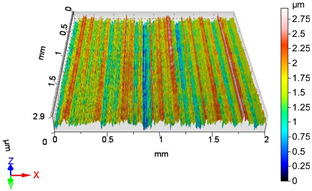	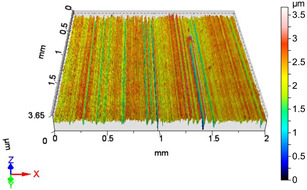
S_a_ = 0.369 µm, S_q_ = 0.457 µm, S_z_ = 3.542 µm, S_p_ = 1.719 µm, S_v_ = 1.823 µm, S_sk_ = −0.059, S_ku_ = 2.739	S_a_ = 0.302 µm, S_q_ = 0.382 µm, S_z_ = 2.678 µm, S_p_ = 1.153 µm, S_v_ = 1.525 µm, S_sk_ = −0.554, S_ku_ = 3.379
V8	**v_2_ = 55.578 m/min, f_2_ = 0.05 mm/rev, F_2_ = 30 N**
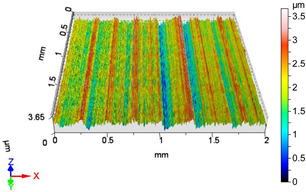	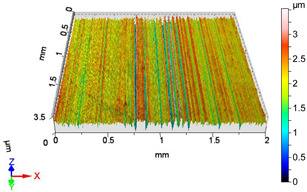
S_a_ = 0.386 µm, S_q_ = 0.478 µm, S_z_ = 3.149 µm, S_p_ = 1.582 µm, S_v_ = 1.567 µm, S_sk_ = 0.210, S_ku_ = 2.802	S_a_ = 0.318 µm, S_q_ = 0.402 µm, S_z_ = 2.849 µm, S_p_ = 1.298 µm, S_v_ = 1.551 µm, S_sk_ = −0.206, S_ku_ = 3.078
V9	**v_1_ = 39.275 m/min, f_1_ = 0.0125 mm/rev, F_3_ = 40 N**
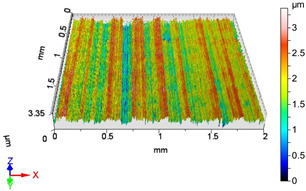	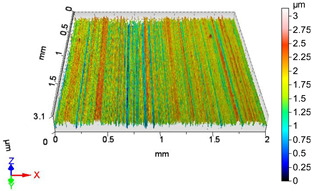
S_a_ = 0.366 µm, S_q_ = 0.454 µm, S_z_ = 3.382 µm, S_p_ = 1.520 µm, S_v_ = 1.862 µm, S_sk_ = −0.082, S_ku_ = 2.803	S_a_ = 0.578 µm, S_q_ = 0.741 µm, S_z_ = 5.242 µm, S_p_ = 2.255 µm, S_v_ = 2.987 µm, S_sk_ = −0.116, S_ku_ = 3.503
V10	**v_2_ = 55.578 m/min, f_1_ = 0.0125 mm/rev, F_3_ = 40 N**
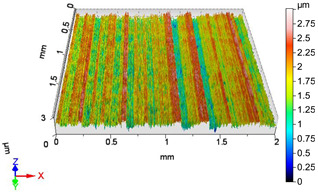	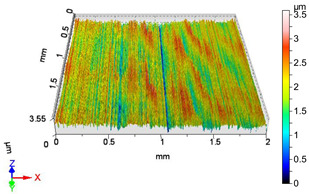
S_a_ = 0.379 µm, S_q_ = 0.466 µm, S_z_ = 3.060 µm, S_p_ = 1.391 µm, S_v_ = 1.669 µm, S_sk_ = 0.022, S_ku_ = 2.609	S_a_ = 0.682 µm, S_q_ = 0.852 µm, S_z_ = 4.988 µm, S_p_ = 2.135 µm, S_v_ = 2.853 µm, S_sk_ = −0.495, S_ku_ = 2.959
V11	**v_1_ = 39.275 m/min, f_2_ = 0.05 mm/rev, F_3_ = 40 N**
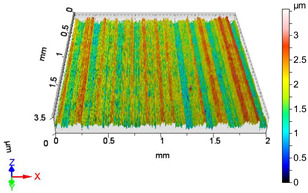	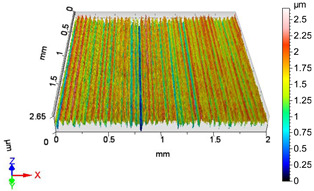
S_a_ = 0.348 µm, S_q_ = 0.435 µm, S_z_ = 2.936 µm, S_p_ = 1.394 µm, S_v_ = 1.542 µm, Ssk = 0.012, S_ku_ = 2.904	S_a_ = 0.289 µm, S_q_ = 0.405 µm, S_z_ = 3.668 µm, S_p_ = 1.405 µm, S_v_ = 2.263 µm, S_sk_ = −1.275, S_ku_ = 7.012
V12	**v_2_ = 55.578 m/min, f_2_ = 0.05 mm/rev, F_3_ = 40 N**
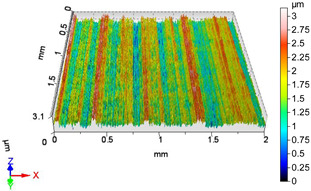	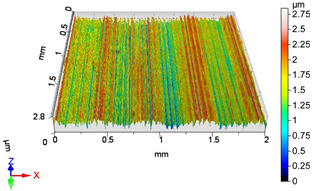
S_a_ = 0.384 µm, S_q_ = 0.492 µm, S_z_ = 3.669 µm, S_p_ = 1.732 µm, S_v_ = 1.937 µm, S_sk_ = −0.069, S_ku_ = 3.147	S_a_ = 0.310 µm, S_q_ = 0.425 µm, S_z_ = 3.526 µm, S_p_ = 1.457 µm, S_v_ = 2.069 µm, S_sk_ = −0.737, S_ku_ = 5.044
V13	**v_1_ = 39.275 m/min, f_1_ = 0.0125 mm/rev, F_4_ = 50 N**
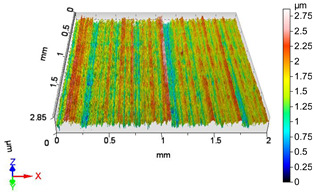	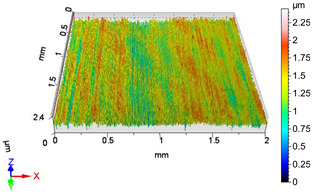
S_a_ = 0.305 µm, S_q_ = 0.385 µm, S_z_ = 2.868 µm, S_p_ = 1.313 µm, S_v_ = 1.555 µm, S_sk_ = −0.084, S_ku_ = 3.148	S_a_ = 0.204 µm, S_q_ = 0.261 µm, S_z_ = 2.447 µm, S_p_ = 1.160 µm, S_v_ = 1.286 µm, S_sk_ = −0.163, S_ku_ = 3.508
V14	**v_2_ = 55.578 m/min, f_1_ = 0.0125 mm/rev, F_4_ = 50 N**
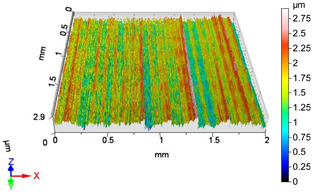	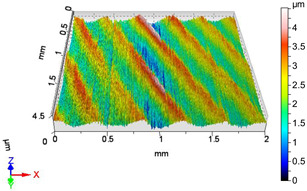
S_a_ = 0.305 µm, S_q_ = 0.388 µm, S_z_ = 2.923 µm, S_p_ = 1.358 µm, S_v_ = 1.565 µm, S_sk_ = −0.021, S_ku_ = 3.290	S_a_ = 0.547 µm, S_q_ = 0.663 µm, S_z_ = 4.521 µm, S_p_ = 2.285 µm, S_v_ = 2.236 µm, S_sk_ = 0.238, S_ku_ = 2.722
V15	**v_1_ = 39.275 m/min, f_2_ = 0.05 mm/rev, F_4_ = 50 N**
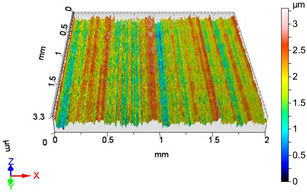	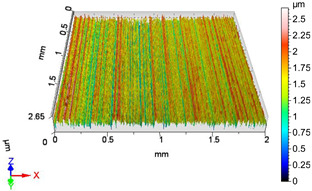
S_a_ = 0.351 µm, S_q_ = 0.439 µm, S_z_ = 3.304 µm, S_p_ = 1.493 µm, S_v_ = 1.812 µm, S_sk_ = −0.027, S_ku_ = 2.915	S_a_ = 0.263 µm, S_q_ = 0.341 µm, S_z_ = 2.680 µm, S_p_ = 1.188 µm, S_v_ = 1.491 µm, S_sk_ = −0.387, S_ku_ = 3.611
V16	**v_2_ = 55.578 m/min, f_2_ = 0.05 mm/rev, F_4_ = 50 N**
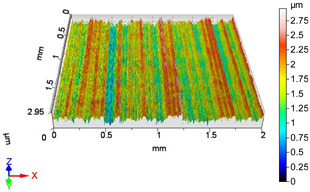	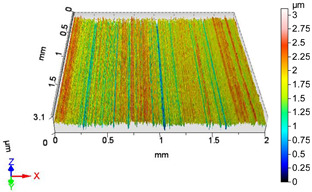
S_a_ = 0.337 µm, S_q_ = 0.419 µm, S_z_ = 2.957 µm, S_p_ = 1.338 µm, S_v_ = 1.691 µm, S_sk_ = -0.019, S_ku_ = 2.777	S_a_ = 0.267 µm, S_q_ = 0.353 µm, S_z_ = 3.115 µm, S_p_ = 1.401 µm, S_v_ = 1.714 µm, S_sk_ = −0.533, S_ku_ = 4.120

## Data Availability

The original contributions presented in this study are included in the article. Further inquiries can be directed to the corresponding author.

## Appendix A. Measurement Data and Improvement

In this appendix, measurement data of arithmetical mean height (*S_a_*), root mean square height (*S_q_*), and maximum height (*S_z_*) are reported for each test specimens before burnishing signed with ’ ((*S_a_’*), (S_q_’), and (S_z_’)) and after burnishing signed without ’ ((*S_a_*), (*S_q_*), and (*S_z_*)). Table A1 contains the improvement ratios *IS_a_*, *IS_q_* and *IS_z_*. The improvement ratios are calculated according to the description given in Equation (1).

**Table A1:** Measured 3D surface roughness parameters for parameter variations V1–V16 before and after burnishing, with calculated improvement ratios.

	Techn. Parameters			Surface Roughness Parameters Before Burnishing			Surface Roughness Parameters After Burnishing			Improvements		
	Speed	Feed	Force									
	v, m/min	f, mm/rev	F, N	S_a_’, µm	S_q_’, µm	S_z_’, µm	S_a_, µm	S_q_, µm	S_z_, µm	*IS_a_* [%]	*IS_q_* [%]	*IS_z_* [%]
V1	39.275	0.0125	20	0.490	0.585	3.277	0.331	0.428	3.414	−32.449	−26.838	4.181
V2	55.578	0.0125	20	0.402	0.493	3.278	0.386	0.486	3.265	−3.980	−1.420	−0.397
V3	39.275	0.0500	20	0.435	0.535	3.400	0.298	0.380	2.817	−31.494	−28.972	−17.147
V4	55.578	0.0500	20	0.446	0.557	3.603	0.258	0.330	2.556	−42.152	−40.754	−29.059
V5	39.275	0.0125	30	0.419	0.563	5.153	0.315	0.400	3.144	−24.821	−28.952	−38.987
V6	55.578	0.0125	30	0.343	0.428	3.019	0.329	0.421	3.588	−4.082	−1.636	18.847
V7	39.275	0.0500	30	0.369	0.457	3.542	0.302	0.382	2.678	−18.157	−16.411	−24.393
V8	55.578	0.0500	30	0.386	0.478	3.149	0.318	0.402	2.849	−17.617	−15.900	−9.527
V9	39.275	0.0125	40	0.392	0.499	3.588	0.578	0.741	5.242	47.449	48.497	46.098
V10	55.578	0.0125	40	0.379	0.466	3.060	0.682	0.852	4.988	79.947	82.833	63.007
V11	39.275	0.0500	40	0.348	0.435	2.936	0.289	0.405	3.668	−16.954	−6.897	24.932
V12	55.578	0.0500	40	0.384	0.492	3.669	0.310	0.425	3.526	−19.271	−13.618	−3.898
V13	39.275	0.0125	50	0.305	0.385	2.868	0.204	0.261	2.447	−33.115	−32.208	−14.679
V14	55.578	0.0125	50	0.305	0.388	2.923	0.547	0.663	4.521	79.344	70.876	54.670
V15	39.275	0.0500	50	0.351	0.439	3.304	0.263	0.341	2.680	−25.071	−22.323	−18.886
V16	55.578	0.0500	50	0.480	0.730	6.568	0.267	0.353	3.115	−44.375	−51.644	−52.573

**Table A2:** Measured volume parameters for parameter variations V1–V16 before and after burnishing.

				Before Burnishing, [ml/m^2^]		After Burnishing, [ml/m^2^]				
	v, m/min	f, mm/rev	F, N	V_mc_’ [ml/m^2^]	V_vc_’, [ml/m^2^]	V_mc_, [ml/m^2^]	V_vc_, [ml/m^2^]	DV_mc_ [ml/m^2^]	DV_vc_ [ml/m^2^]	DV_mc_/DV_vc_
V1	39.275	0.0125	20	0.586	0.722	0.369	0.444	−0.217	−0.278	0.781
V2	55.578	0.0125	20	0.471	0.586	0.434	0.618	−0.037	0.032	−1.156
V3	39.275	0.0500	20	0.500	0.646	0.336	0.403	−0.164	−0.243	0.675
V4	55.578	0.0500	20	0.513	0.670	0.290	0.349	−0.223	−0.321	0.695
V5	39.275	0.0125	30	0.448	0.620	0.365	0.453	−0.083	−0.167	0.497
V6	55.578	0.0125	30	0.392	0.521	0.368	0.464	−0.024	−0.057	0.421
V7	39.275	0.0500	30	0.427	0.566	0.347	0.408	−0.080	−0.158	0.506
V8	55.578	0.0500	30	0.439	0.611	0.354	0.473	−0.085	−0.138	0.616
V9	39.275	0.0125	40	0.441	0.545	0.646	0.905	0.205	0.360	0.569
V10	55.578	0.0125	40	0.440	0.588	0.805	0.912	0.365	0.324	1.127
V11	39.275	0.0500	40	0.393	0.537	0.292	0.361	−0.101	−0.176	0.574
V12	55.578	0.0500	40	0.422	0.593	0.322	0.412	−0.100	−0.181	0.552
V13	39.275	0.0125	50	0.342	0.454	0.227	0.300	−0.115	−0.154	0.747
V14	55.578	0.0125	50	0.341	0.453	0.601	0.846	0.260	0.393	0.662
V15	39.275	0.0500	50	0.396	0.539	0.291	0.377	−0.105	−0.162	0.648
V16	55.578	0.0500	50	0.451	0.633	0.284	0.382	−0.167	−0.251	0.665

**Table A3:** Measured volume parameters for parameter variations F1–F8 before and after burnishing.

				Before Burnishing [mL/m^2^]				After Burnishing [mL/m^2^]			
	v, m/min	f, mm/rev	F, N	V_mp_, [ml/m^2^]	V_mc_, [ml/m^2^]	V_vc_, [ml/m^2^]	V_vv_, [ml/m^2^]	V_mp_, [ml/m^2^]	V_mc_, [ml/m^2^]	V_vc_, [ml/m^2^]	V_vv_, [ml/m^2^]
F1	39.275	0.1	20	0.058	1.365	1.836	0.134	0.020	0.852	0.786	0.154
F2	39.275	0.1	30	0.051	1.359	1.745	0.142	0.017	0.636	0.686	0.146
F3	39.275	0.1	40	0.059	1.341	1.747	0.123	0.020	0.455	0.573	0.116
F4	39.275	0.1	50	0.051	1.247	1.657	0.116	0.020	0.422	0.570	0.098
F5	55.578	0.1	20	0.047	1.266	1.776	0.110	0.022	0.767	0.766	0.147
F6	55.578	0.1	30	0.065	1.401	1.855	0.120	0.020	0.560	0.604	0.139
F7	55.578	0.1	40	0.064	1.308	1.860	0.135	0.015	0.372	0.418	0.112
F8	55.578	0.1	50	0.064	1.274	1.736	0.115	0.016	0.303	0.387	0.078

## Appendix B

An ANOVA analysis is presented for final models in the case of the improvement ratios of *IS_a_*, *IS_q_*, and *IS_z_* (Table A4, Table A5, and Table A6, respectively) and data.log normalization (Table A7, Table A8 and Table A9).

**Table A4:** Dependent variable: *IS_a_*, ANOVA output.

Source	df	Mean Square	F	Sig	Partial Eta Squared
Corrected Model	5	2751.96	2.772	0.08	0.581
Intercept	1	712.863	0.718	0.417	0.067
v m/min	1	1648.888	1.661	0.227	0.142
f mm/rev	1	6536.076	6.583	0.028	0.397
F N	3	1858.279	1.872	0.198	0.360
Error	10	992.907			
Total	16				
Corrected Total	15				

R Squared = 0.581 (Adjusted R Squared = 0.371).

The model explains 58.1% of the variance in IS_a_. The only statistically significant factor is feed (f); speed (v) and force (F) show meaningful effect sizes.

**Table A5:** Dependent variable: *IS_q_* ANOVA output.

Source	df	Mean Square	F	Sig	Partial Eta Squared
Corrected Model	5	2701.375	2.906	0.071	0.592
Intercept	1	455.470	0.490	0.500	0.047
v m/min	1	1275.222	1.372	0.269	0.121
f mm/rev	1	5916.340	6.365	0.030	0.389
F N	3	2105.104	2.265	0.143	0.405
Error	10	929.571			
Total	16				
Corrected Total	15				

R Squared = 0.592 (Adjusted R Squared = 0.371).

The model explains 58.1% of the variance in *IS_q_*. Similarly, the feed is dominating *IS_q_*; however, speed and force have a minor effect.

**Table A6:** Dependent variable: *IS_z_* ANOVA output.

Source	df	Mean Square	F	Sig	Partial Eta Squared
Corrected Model	5	2079	3.259	0.053	0.620
Intercept	1	0.299	0	0.983	0
v m/min	1	399.5	0.626	0.447	0.059
f mm/rev	1	4333	6.792	0.026	0.404
F N	3	1887	2.958	0.084	0.470
Error	10	637.9			
Total	16				
Corrected Total	15				

R Squared = 0.620 (Adjusted R Squared = 0.430).

The model explains 62% of the variance in *IS_z_*, and the feed is dominating factor.

The data was transformed using logarithmic transformation to reduce the skewness. ANOVA analysis was performed for each variable before and after burnishing. Table A7 presents the ANOVA analysis for the arithmetical mean height surface roughness parameter before (*S_a_’*) and after (*S_a_*) burnishing and the effect of the machining parameter to define the significance of the burnishing. Similarly, Table A8 presents the ANOVA analysis for root mean square height surface roughness parameter before (*S_q_’*) and after (*S_q_*) burnishing and the effect of the machining parameter to define the significance of the burnishing. Furthermore, Table A9 relates to using logarithmic transformation of maximum height before (*S_z_’*) and after (*S_z_*) burnishing.

**Table A7:** ANOVA results after normalization of data: *S_a_’*, *S_a_*.

Dependent Variable: *S_a_*’					Dependent Variable: *S_a_*				
Source	df	Mean Square	F	Sig	Source	df	Mean Square	F	Sig
Corrected Model	5	0.004	1.325	0.329	Corrected Model	5	0.118	5.661	0.01
Intercept	1	2.429	901.136	0	Intercept	1	23.015	1100.347	0
v m/min	1	1.60 × 10^−5^	0.006	0.940	v m/min	1	0.033	1.581	0.237
f mm/rev	1	0.002	0.624	0.448	f mm/rev	1	0.206	9.828	0.011
F N	3	0.005	1.998	0.178	F N	3	0.118	5.633	0.016
Error	10	0.003			Error	10	0.021		
Total	16				Total	16			
Corrected Total	15				Corrected Total	15			
R Squared = 0.398 (Adjusted R Squared = 0.098)					R Squared = 0.739 (Adjusted R Squared = 0.608)				

*S_a_’* before burnishing the model is not significant; after burnishing, the model is significant and R^2^ is strong. After burnishing, feed and force strongly influence *S_a_*.

**Table A8:** ANOVA results after normalization of data: *S_q_’*, *S_q_*.

Dependent Variable: *S_q_*’					Dependent Variable: *S_q_*				
Source	df	Mean Square	F	Sig	Source	df	Mean Square	F	Sig
Corrected Model	5	0.004	0.425	0.821	Corrected Model	5	0.048	3.352	0.049
Intercept	1	3.930	430.220	0	Intercept	1	3.303	231.201	0
v m/min	1	0.001	0.123	0.733	v m/min	1	0.022	1.543	0.242
f mm/rev	1	0.006	0.683	0.428	f mm/rev	1	0.095	6.661	0.027
F N	3	0.004	0.439	0.730	F N	3	0.041	2.852	0.091
Error	10	0.009			Error	10	0.014		
Total	16				Total	16			
Corrected Total	15				Corrected Total	15			
R Squared = 0.175 (Adjusted R Squared = −0.237)					R Squared = 0.626 (Adjusted R Squared = 0.439)				

Similarly, for *S_q_*, the model is significant after burnishing, and feed rate is the most dominating factor influencing *S_q._*

**Table A9:** ANOVA results after normalization of data: *S_z_’*, *S_z_*.

Dependent Variable: *S_z_*’					Dependent Variable: *S_z_*				
Source	df	Mean Square	F	Sig	Source	df	Mean Square	F	Sig
Corrected Model	5	0.322	0.263	0.923	Corrected Model	5	1.607	5.898	0.009
Intercept	1	205.471	168.153	0	Intercept	1	185.627	681.065	0
v m/min	1	0.09	0.074	0.791	v m/min	1	0.336	1.232	0.293
f mm/rev	1	0.564	0.462	0.512	f mm/rev	1	2.822	10.355	0.009
F N	3	0.318	0.260	0.852	F N	3	1.626	5.967	0.013
Error	10	1.222			Error	10	0.273		
Total	16				Total	16			
Corrected Total	15				Corrected Total	15			
R Squared = 0.116 (Adjusted R Squared = −0.325)					R Squared = 0.747 (Adjusted R Squared = 0.620)				

After burnishing, the *S_z_* model is highly significant. Feed and force show strong significance on *S_z_*.
